# Deep Brain Stimulation and Sleep-Wake Disturbances in Parkinson Disease: A Review

**DOI:** 10.3389/fneur.2018.00697

**Published:** 2018-08-27

**Authors:** Vibhash D. Sharma, Samarpita Sengupta, Shilpa Chitnis, Amy W. Amara

**Affiliations:** ^1^Department of Neurology, University of Kansas Medical Center, Kansas City, KS, United States; ^2^Department of Neurology, University of Southwestern Medical Center, Dallas, TX, United States; ^3^Department of Neurology, University of Alabama at Birmingham, Birmingham, AL, United States

**Keywords:** sleep-wake disturbances, deep brain stimulation, subthalamic nucleus, Parkinson disease, sleep-wake pathophysiology

## Abstract

Sleep-wake disturbances are common non-motor manifestations in Parkinson Disease (PD). Complex pathophysiological changes secondary to neurodegeneration in combination with motor symptoms and dopaminergic medications contribute to development of sleep-wake disturbances. The management of sleep complaints in PD is important as this symptom can affect daily activities and impair quality of life. Deep brain stimulation (DBS) is an effective adjunctive therapy for management of motor symptoms in PD. However, its effect on non-motor symptoms including sleep-wake disturbances is not widely understood. In this article, we reviewed studies assessing the effect of DBS at various therapeutic targets on sleep-wake disturbances. Of the studies examining the role of DBS in sleep-wake disturbances, the effect of subthalamic nucleus stimulation is most widely studied and has shown improvement in sleep quality, sleep efficiency, and sleep duration. Although, studies investigating changes in sleep with stimulation of thalamus, globus pallidus interna, and pedunculopontine nucleus are limited, they support the potential for modulation of sleep-wake centers with DBS at these sites. The mechanism by which DBS at different anatomical targets affects sleep-wake disturbances in PD is unclear and may involves multiple factors, including improved motor symptoms, medication adjustment, and direct modulation of sleep-wake centers.

## Introduction

Parkinson disease (PD) is a complex neurodegenerative disorder that leads to both motor and non-motor symptoms. The cardinal motor symptoms of PD include bradykinesia, rigidity, tremor, and gait difficulty. Non-motor manifestations include sleep disorders, neuropsychiatric symptoms, autonomic dysfunction, and cognitive decline (1). Conventionally, the management of PD was focused on motor symptoms and non-motor symptoms were often neglected. Over the last few decades, non-motor symptoms have gained more attention for their significant negative impact on quality of life in PD, leading to more exploration of how the neurodegenerative process influences these symptoms (2, 3).

Sleep-wake disturbances are a common non-motor symptom associated with PD and were first described by James Parkinson in his original article “Essay on shaking palsy” (4). Patients with PD can experience multiple sleep disorders, including sleep fragmentation, rapid eye movement (REM) sleep behavior disorder (RBD), excessive daytime sleepiness (EDS), periodic limb movements of sleep (PLMS), and restless legs syndrome (RLS) (5). Sleep-wake disturbances in PD contribute to poor quality of life, impaired mood and behavior, and increased morbidity and mortality (3, 6, 7). Due to the limited treatment options for sleep disorders in PD, management of these symptoms can be challenging.

Deep brain stimulation (DBS) effectively treats the motor symptoms of PD, as well as improving motor fluctuations and quality of life (8–10). However, the effects of DBS therapy on non-motor symptoms, including sleep-wake disturbances, have received less attention. The available evidence suggests that DBS therapy can impact different aspects of sleep. We conducted a literature search on PubMed to identify studies evaluating the effects of DBS on either subjective or objective sleep parameters in PD. The following keywords were used in different combination for searching articles: “Deep brain stimulation,” “Parkinson disease,” “sleep disturbances,” “sleep quality,” “REM sleep behavior disorder,” “restless legs syndrome,” “excessive daytime sleepiness,” subthalamic nucleus, “globus pallidus interna,” “ventral intermediate nucleus,” “pedunculopontine nucleus,” “sleep pathophysiology.” Both prospective and retrospective studies published in English language between 2000 and 2017 were reviewed. Also, relevant review articles on sleep disorders and Parkinson disease were reviewed. This article will briefly discuss pathophysiology of sleep-wake disturbances in PD; review existing literature exploring the effects of DBS at different therapeutic targets on sleep-wake disturbances, highlight the gaps in our understanding, and offer insight into potential future directions of investigating DBS as therapy for managing PD-related sleep disorders.

## Results

### Pathophysiology of sleep-wake disturbances in parkinson disease

The pathophysiological processes underlying sleep-wake disturbances in PD are complex, with influences from multiple factors such as neurochemical changes secondary to the neurodegenerative process, motor symptoms, autonomic dysfunction, and medication-induced alterations of sleep architecture and the circadian rhythm.

Dopaminergic neurons projecting to the striatum, cortex, and limbic structures play a significant role in regulation of the sleep-wake cycle (11). In PD, there is a gradual loss of dopaminergic neurons in the substantia nigra pars compacta and the ventral tegmental area (VTA) (12, 13). Loss of dopamine in mesocorticolimbic and mesostriatal pathways changes thalamocortical rhythms, resulting in impaired regulation of REM sleep, excessive daytime sleepiness, reduced deep sleep, and reduced sleep efficiency (11, 13, 14).

In addition, dysregulation of non-dopaminergic subcortical pathways from degenerative changes in the noradrenergic locus ceruleus, serotonergic raphe nucleus, and the cholinergic nucleus of Meynert and pedunculopontine nucleus (PPN) also play an important role in the development of sleep disorders (12, 15, 16). The PPN modulates wakefulness and REM sleep through its cholinergic inputs to the thalamic nuclei, particularly the relay nuclei and the reticular nucleus. Cholinergic inputs to these nuclei modulate firing patterns of thalamocortical and reticular thalamic neurons, thereby controlling transitions between wakefulness and sleep. PPN also provides input to orexin/hypocretin neurons located in hypothalamus and forebrain nucleus basalis, which regulate the sleep-wake cycle and promote arousal and attention (17). Some studies suggest that patients with PD may have loss of hypocretin in the brain and CSF, although other studies did not replicate this finding (18, 19). Loss of PPN neurons is implicated in sleep disorders as well as gait difficulty in PD (17).

Other factors contributing to sleep disturbances in PD patients can include nocturia, night-time cramps, motor symptoms including rigidity, tremor, dystonia, bradykinesia, and back pain (20–22). Nocturia is a common non-motor symptom in PD and is associated with increased nocturnal activity contributing to sleep maintenance insomnia (23). On polysomnography, PD patients with nocturia were shown to have reduced sleep efficiency and total sleep time (21). Motor symptoms such as bradykinesia, rigidity, and tremor occur frequently in PD patients with wearing off of dopaminergic medications at night. These symptoms impair bed mobility, interrupt sleep, and decrease sleep efficiency (24). Although dopaminergic medications can improve motor symptoms and therefore potentially improve sleep, they can also be associated with poor sleep quality, decreased REM sleep, and excessive daytime sleepiness, including unexpected sudden onset of sleep (sleep attacks) (25–27).

### DBS in parkinson disease

DBS is an established therapy for motor complications in PD. The procedure involves surgical implantation of electrodes in specific brain regions. These electrodes are connected to a neurostimulator, which provides electrical impulses to the targeted areas and modulates brain circuits. The two commonly targeted sites for treating the cardinal motor symptoms of PD are subthalamic nucleus (STN) and globus pallidus interna (Gpi) (28). Other targets such as ventral intermediate nucleus (VIM) of thalamus are less often utilized. Multiple clinical trials have shown that high frequency DBS therapy can be superior to best medical therapy for motor complications in PD (8–10, 29). Despite clear efficacy of DBS in PD, its mechanism is not well understood and different hypotheses have been proposed (30, 31). Although, discussion of these hypotheses is beyond the scope of this article, it is suggested that DBS, through its excitatory and inhibitory effects on the targeted nucleus, disrupts abnormal information within cortico-basal ganglia-thalamic neural circuits, which results in improvement of motor symptoms (30, 31).

### DBS and sleep-wake disturbances in PD

The mechanism by which stimulation at different targets such as STN, GPi, and PPN regulates sleep architecture is not clearly understood. Effect of DBS on sleep disturbances may depend on the site of stimulation. STN has projections to sleep-regulatory centers including the thalamus, PPN, and cortex (32). Local field potential recorded from STN during sleep have shown significant differences in band-power across different stages of sleep (33), suggesting a role in the sleep regulatory network. GPi is an important output nucleus of the basal ganglia which also has projections to sleep-wake modulating centers including the thalamus and PPN. Although the exact mechanism is unknown, it is possible that DBS at these sites modulates the sleep-wake network and directly affects sleep physiology. Both GPi and STN project to the globus pallidus externa (GPe), and it is suggested that improvement in sleep disturbances after DBS at these sites could be regulated through GPe (34).

The role of DBS in sleep-wake dysfunction in PD has been analyzed in multiple studies. These studies have utilized subjective and/or objective sleep measures (see below) to assess sleep outcomes after DBS surgery.

### Measures of sleep parameters

Subjective sleep outcomes are measured with questionnaires, while objective sleep measures include polysomnography (PSG) and actigraphy. Commonly used scales for assessing subjective sleep include Parkinson Disease Sleep Scale (PDSS), PDSS-2, Pittsburgh Sleep Quality Index (PSQI), and Epworth Sleepiness Scale (ESS). PDSS is a validated self-rating scale consisting of 15 questions that quantify different aspects of sleep, including overall quality of nighttime sleep (item 1), sleep onset and sleep maintenance insomnia (items 2 and 3), nocturnal restlessness (items 4 and 5), distressing dreams (item 6), nocturnal psychosis (item 7), nocturia (items 8 and 9), nocturnal motor and sensory-motor symptoms (items 10–13), unsatisfactory sleep refreshment (item 14), and daytime dozing (item 15). Each question is rated on a visual analog scale from 0 (severe and always present) to 10 (not present) and scores are added. Higher scores indicate better sleep quality (35). PDSS-2 is a revised version of PDSS with more items on different aspects of sleep disturbances, such as restless legs syndrome and sleep apnea, and inclusion of a measure of frequency of symptoms (36). PSQI is a self-rated questionnaire that assesses sleep quality and disturbances over a 1 month interval. Scores are generated based on seven components assessing subjective sleep quality, sleep latency, sleep duration, habitual sleep efficiency, sleep disturbances, use of sleeping medication, and daytime dysfunction. Total scores range from 0 to 21 and a score >5 indicates impaired sleep quality (37). ESS is a self-rating scale with 8 questions (scored from 0 to 3) that assesses likelihood of falling asleep in different situations, such as reading or driving. The total scores range from 0 to 24 with a score >10 indicating excessive daytime sleepiness (38).

#### Subthalamic nucleus (STN) DBS and sleep-wake disturbances

The effect of STN DBS on sleep-wake disturbances has been examined in multiple studies (Table 1). Bauman-Vogel and colleagues studied 50 PD patients before and 6 months after bilateral STN DBS with Zurich sleep questionnaire, ESS, actigraphy, and PSG to assess both subjective and objective sleep parameters. They found that post STN DBS, self-reported mean sleep duration increased by around 40 mins (*p* < 0.001) and EDS reduced by 2.1 points (*p* < 0.001) on ESS. Improvement in total sleep time was also confirmed by actigraphy recording (improvement of ~1 h, *p* < 0.001) and PSG (improvement by 21.2 min, *p* = 0.016). PSG results showed significant increase in sleep efficiency and deep sleep, and a reduction in wake after sleep onset (WASO) and REM latencies. PLMS indices doubled with stimulation, which was associated with reduction in dose of dopamine agonists. No significant change was seen in sleep fragmentation, occurrence of RBD and prevalence of RLS (39).

**Table 1:** Summary of studies examining the effects of STN DBS on sleep.

**Study**	***N***	**Study type**	**Outcome measures**	**Outcome**
Bauman-Vogel et al. (39)	50	Prospective, 6 mo	Zurich sleep questionnaire ESS Actigraphy recording PSG	Significant improvement in sleep duration & sleep efficiency Significant decrease in WASO, REM latency No change in sleep fragmentation, RBD and RLS
Amara et al. (40)	20	Cross sectional	PSG- DBS high freq vs. low freq	No significant difference in sleep parameters
Tolleson et al. (41)	5	Prospective, 6 mo	PSG	Reduced WASO, sleep latency and REM latency
Marques et al. (42)	31	Prospective, 6 mo	RLS	Emergence of RLS in 6 pts
Breen et al. (43)	11	Prospective, 6 mo	PDSS	Significant improvement in sleep quality
Deli et al. (44)	13	Prospective, 12 mo	PDSS-2	Significant improvement in sleep quality
Amara et al. (45)	53	Prospective, 6 mo	PSQI	Significant improvement in sleep quality
Nishida et al. (46)	10	Prospective, NR	PSG PDSS	Significant decrease in WASO Decrease in REM sleep without atonia and increase in normal REM sleep Improved subjective sleep quality
Chahine et al. (47)	17	Prospective, 6 mo	PDSS ESS IRLSSG scale	Significant improvement in sleep quality, ESS & RLS scores
Driver-dunckley et al. (48)	6	Retrospective	RLS	Significant improvement in RLS scores (improved by 84%) Mean LED reduction 56%
Lyons et al. (49)	43	Retrospective	Patient diaries ESS	Significant improvement in sleep quality, early morning dystonia No change in excessive daytime sleepiness
Hjort et al. (50)	10	Prospective, 3 mo	PDSS	Significant improvement in sleep quality No change in EDS and nocturia
Cicolin et al. (51)	5	Prospective, 3 mo	PSG	Significant reduction in WASO significant, increase in sleep efficiency, reduced REM latency No change in REM and PLMS
Monaco et al. (52)	10	Prospective, 3 mo	PSQI PSG—DBS ON vs. OFF	Significant improvement in subjective sleep quality ON DBS—Significant improvement in total sleep time, efficiency and duration of slow wave and REM sleep
Kedia et al. (53)	195	Retrospective	RLS	Emergence of RLS in 11 pts Mean LED reduction 74%
Iranzo et al. (54)	11	Prospective, 6 mo	PSQI PSG ESS	Significant improvement in subjective sleep quality Significant increase in continuous sleep, reduction in the arousal index and increase in nocturnal mobility There was no change in RBD and PLM increased after surgery
Arnulf et al. (55)	10	Prospective, 3 mo	PSG—DBS ON and OFF	ON DBS—Significant improvement in sleep duration, sleep efficiency, stage II sleep. Decrease in WASO No significant change in sleep fragmentation, RBD PLM worse with DBS ON

*DBS, Deep Brain Stimulation; BL, bilateral; Uni, unilateral; PSG, Polysomnography; PSQI, Pittsburgh Sleep Quality Index; PDSS, Parkinson Disease Sleep Scale; WASO, wake time after sleep onset; ESS, Epworth Sleepiness scale; IRLSSG, International RLS Study Group; RBD, Rapid Eye Movement Sleep Behavior Disorder; LED, Levodopa equivalent dose*.

Multiple other studies have analyzed effect of STN DBS on sleep parameters. Nishida and colleagues evaluated 10 patients (bilateral STN 8, unilateral 2) with PDSS and PSG at 1 week prior to DBS surgery and 1 week post-DBS programming. They found significant improvement in subjective sleep quality and excessive sleepiness (*p* = 0.01) after DBS. PSG results showed a significant decrease in WASO and REM sleep without atonia and an increase in normal REM sleep duration after DBS (*p* = 0.03) (46). Another study evaluated 5 PD patients with bilateral STN using PSG and found significant reduction in WASO (−48%), latency to the first REM period (−58%), and significant increase in sleep efficiency (+60%) after 3 months of DBS. Total sleep time improved by 33% but this was not statistically significant. No changes in PLMS and RBD were observed (51). Iranzo and colleagues studied sleep outcomes in 11 PD patients with bilateral STN DBS. PSQI, ESS and PSG were done before and at 6 months post-DBS. They reported a significant improvement in PSQI scores (9.4 points) post-DBS. Improvement was seen in sleep quality, latency and sleep duration. PSG showed significant increase in continuous sleep, reduction in the arousal index and increase in nocturnal mobility. There was no change in RBD and PLMS index increased after surgery (54).

Few studies compared results of ON and OFF DBS stimulation on sleep-wake disturbances. Monaco and colleagues studied 10 patients with bilateral STN DBS and compared sleep parameters pre- and post-DBS (OFF and ON stimulation) at 3 months using PSQI questionnaire and PSG. The study found a significant improvement in subjective sleep quality (mean reduction of PSQI scores by 6.4 points) after surgery. There was a significant improvement in total sleep time (+ ~80 mins), sleep efficiency, and duration of slow wave and REM sleep (*p* < 0.01) compared to pre-DBS evaluations. The difference was not seen when DBS was turned OFF. As none of the patients had PLMS and RBD pre- and post-DBS, these outcomes were not analyzed (52). In another study, Arnulf and colleagues evaluated 10 PD patients with PSG under OFF and ON stimulation conditions after 3–6 months of bilateral STN DBS. PSG was done during OFF and ON stimulation in two night sessions. A significant increase in sleep duration (47%), sleep efficiency (36%), stage 2 sleep (63%) and decrease in WASO (−51 mins) was seen ON stimulation compared to OFF stimulation. There were no significant differences in sleep fragmentation or RBD symptoms between ON and OFF stimulation. PLM indices were less frequent during OFF stimulation (55).

In another study, Amara and colleagues studied differential effects of alternate DBS frequencies on sleep measures in 20 patients with STN DBS (18 unilateral, 2 bilateral). Patients underwent PSG on 3 non-consecutive nights with DBS OFF, DBS at high frequency (≥130 Hz), and DBS at low frequency (60 Hz). The authors did not see a significant difference in sleep efficiency or other sleep parameters between the two frequencies. A trend toward improvement was seen in total sleep time with stimulation at higher frequency and a shorter REM latency was seen with stimulation at lower frequency compared to OFF DBS (40). Surprisingly, sleep was not better with DBS ON compared to DBS OFF in many of the participants, suggesting that some patients may benefit from adaptive stimulation during which stimulation could be altered in different behavioral states (wake and sleep). Effects of microsubthalamotomy on sleep measures after bilateral STN DBS (*n* = 15) was evaluated in another study, which showed a significant improvement in sleep quality (*P* < 0.001) with participants reporting longer total sleep duration, decreased daytime sleepiness, and improvement in RLS symptoms in the immediate post-operative period prior to turning on DBS. PSG data showed an increase in total sleep time and sleep efficiency with a decrease in WASO and arousal index (56).

Additional studies utilized subjective sleep parameters as the primary outcome and showed significant improvement in sleep quality with STN DBS. Deli and colleagues measured PDSS-2 scores in 13 participants with PD related sleep complaints before and after STN DBS and found significant improvement in quality of sleep at 1 year (*p* < 0.001) (44). Hjort and colleagues assessed subjective sleep quality with PDSS questionnaire in 10 patients with bilateral STN DBS and compared results to controls who did not have DBS. They found a significant improvement in PDSS scores in the DBS group at 3 months compared to pre-DBS and controls. Significant improvement was seen in overall quality of sleep and nocturnal motor symptoms. There was no change in nocturia, sleep fragmentation, or daytime sleepiness (50). Another study assessing subjective sleep quality with PDSS also reported significant improvement in sleep parameters following STN DBS (43). In a study using the PSQI questionnaire, the effects of unilateral STN DBS on subjective sleep quality was examined in 53 consecutive PD patients before and after unilateral STN DBS. The study found that unilateral STN DBS significantly improved subjective sleep quality at 6 months compared to pre-DBS baseline (*p* = 0.013). PSQI sub-scores including sleep quality and sleep disturbances significantly improved (*p* < 0.01), while sleep latency, sleep duration, sleep efficiency, use of sleep medications, and daytime dysfunction showed a trend toward improvement (45). In a long-term study in 43 PD patients, bilateral STN DBS was shown to increase total sleep time and reduce patient reported sleep problems and early morning dystonia for up to 24 months. There was no change in excessive daytime sleepiness (49).

Studies on the effects of STN DBS on restless legs symptoms have conflicting results. Chahine and colleagues studied 17 PD patients with STN DBS and found significant improvement in International Restless Legs Syndrome Study Group (IRLSGG) rating scale scores at 6 months in six patients (−9.2, *p* = 0.037) as well as improvement in sleep quality and excessive sleepiness (47). A retrospective study also showed an improvement of 84% in IRLSGG scale scores after bilateral STN DBS. This improvement was despite a mean reduction in levodopa equivalent dose (LED) by 56% (48). On the contrary, a study by Kedia and colleagues reported emergence of RLS in 11 of 195 patients post-DBS surgery. The mean reduction in LED was 74% in patients who developed RLS compared to 40% reduction in those who did not develop RLS. This study did not use rigorous diagnostic criteria for RLS so some patients may have been misdiagnosed (53). Another study aimed to identify factors associated with development of RLS after STN DBS. The study found that, of 31 total participants, six patients who were originally free from RLS symptoms had emergence of RLS symptoms at 6 months after DBS. Interestingly, patients with emergence of RLS had a significantly higher dose of dopamine agonists post-DBS (mean 155 mg/day) compared to PD patients without emergence of RLS (mean 0.00 mg/day) (*p* = 0.043) and a lower percentage change in dopamine agonist treatment in RLS group compared to patients without RLS (0.00 vs. 66.67%, *p* = 0.043). The authors concluded that overstimulation resulting from cumulative effects of dopamine agonists and STN DBS may lead to emergence of RLS (42).

#### Globus pallidus internus (GPi) DBS and sleep-wake disturbances

There is limited data on effect of GPi DBS on sleep-wake disturbances in PD. The only study analyzing effects of GPi DBS on objective sleep outcomes found improvement in sleep quality and efficiency, and decreased WASO, sleep latency and REM latency in 5 PD patients at 6 months. These improvements were not statistically significant (41). Other studies have reported improvement in subjective daytime sleepiness and sleep quality; however, in these studies, sleep-related parameters were not the primary outcome measures (57, 58).

In a randomized clinical trial comparing results GPi vs. STN DBS (NSTAPS) on clinical outcomes up to 3 years after surgery, subjective sleep quality was assessed with PDSS. The study reported that with bilateral GPi stimulation there was an improvement in PDSS scores at 12 months (+7.2, *n* = 62) and 36 months (+ 12.1, *n* = 47) compared to baseline. No significant difference was seen in PDSS scores between STN and GPi groups (59).

#### Role of other DBS targets in sleep-wake disturbances

PPN is an important modulator of sleep-wake cycle. Few studies have investigated the effect of PPN stimulation on objective or subjective sleep parameters in PD. The influence of PPN stimulation on sleep was first reported in a single patient who underwent DBS placement in bilateral STN and PPN. PSG measures pre- and post-DBS with STN-ON and PPN-ON were compared. Both bilateral STN DBS and PPN DBS improved sleep efficiency and decreased WASO and nocturnal awakenings. Surprisingly, PPN DBS at 25 Hz significantly increased REM sleep duration, which was not seen with STN DBS (60). The same group assessed effect of PPN DBS on subjective sleep quality measures using ESS, PDSS and PSQI in six patients with bilateral STN and PPN DBS. Analysis was done under three circumstances during which STN DBS was kept ON: PPN-OFF, continuous PPN-ON, and cyclic PPN-ON. The duration of the study was not reported. Stimulation parameters were kept the same for 2 weeks prior to assessment. The authors found a significant improvement in daytime sleepiness with both continuous and cyclic PPN DBS and improvement in daytime sleepiness, nocturnal restlessness, and psychosis with cyclic PPN DBS. In this study another patient had PSG, which showed that PPN DBS (25 Hz) improved sleep efficiency, decreased awakenings, and significantly increased REM sleep (61). This group later evaluated long-term effects of PPN DBS on the subjective sleep quality in five patients using PDSS and ESS scales. Evaluations were done pre-DBS and post-DBS at 3 months and 12 months. During the study duration STN DBS was kept continuously ON, and PPN DBS was investigated under three conditions; STN-ON (185 Hz), PPN-ON (25 Hz), and PPN-cyclic (25 Hz) during which PPN was kept ON only at night for 12 h. Each stimulation parameter was kept for 2 weeks prior to assessment. All patients reported poor sleep quality prior to DBS. Post-DBS at 3 months there was a significant improvement in nocturnal sleep quality under all DBS conditions, with a mean improvement in PDSS global score by 41% in STN-ON (*P* < 0.05), 35% in PPN-ON (*P* < 0.05), and 57% in PPN-cyclic (*P* < 0.05). PPN DBS also improved sleep onset and maintenance insomnia compared to STN DBS, while PPN-cyclic improved nocturnal restlessness, psychosis and dozing compared to PPN-ON continuous and STN DBS. Sleep quality measures further improved at 12 months with PPN DBS. Excessive daytime sleepiness significantly improved at 3 months with only PPN-ON (46%; *P* < 0.05) and PPN-cyclic (60%; *P* < 0.05), and this improvement persisted at 12 months. STN DBS did not improve ESS score at 3 and 12 months (62).

Another study by Lim et al. studied effect of unilateral PPN DBS on PSG in five patients with Parkinsonism. The frequency of PPN DBS was 70 Hz in three patients with PD and between 5 and 30 Hz in two patients with Progressive supranuclear palsy (PSP). Assessments were done in both PPN-OFF and PPN-ON state up to 12 months after surgery. The study found a significant increase in REM sleep with PPN-ON as compared to PPN-OFF (*p* = 0.03) and WASO improved with PPN-ON (*p* = 0.21). Sleep duration and non-REM sleep remained unchanged. There was no change in RBD in two patients who had RBD (63).

PPN DBS was also found to have effect on alertness. A study involving two patients with STN DBS who subsequently underwent bilateral PPN DBS, showed that high-frequency PPN stimulation induces sleep. These findings were supported by results of daytime PSG study under different conditions including OFF stimulation, and left, right, and bilateral PPN stimulation at either low or higher frequencies. The authors found that high frequency stimulation (80 Hz) induced sleep and low frequency stimulation (10–25 Hz) enhanced alertness. In one patient sudden withdrawal of low-frequency stimulation consistently induced sleep within 0.6–1.7 min and REM sleep within 3–6 min (64).

The role of high frequency VIM DBS on sleep architecture was evaluated in one study. In this study six patients (4 PD and 2 ET) underwent PSG study during VIM OFF and ON DBS. No significant difference was noted in sleep spindle or architecture between OFF and ON DBS. Low frequency stimulation, either continuous or cyclic, in region of the reticular nuclei did not induce sleep in awake patients (65).

## Discussion

DBS therapy revolutionized the management of motor symptoms in PD and has become a widely accepted treatment option. However, its effect on non-dopaminergic symptoms such as neuropsychiatric issues, autonomic dysfunction, and sleep disturbances is not entirely clear and newer targets are being investigated (66). As non-motor symptoms have significant impact on quality of life in patients with PD, developing new treatments or understanding the impact of established treatments such as DBS on these symptoms is important. With the neuromodulatory potential of DBS on different neural circuits, its effect on non-dopaminergic symptoms including sleep-wake disturbances with stimulation at different targets have been studied.

The current evidence suggest that DBS therapy improves different aspects of sleep-wake disturbances in PD. Studies analyzing effects of STN DBS on both subjective and objective sleep parameters have shown a significant improvement in sleep quality, sleep efficiency and sleep duration (39, 46, 52, 54). The duration of WASO and REM latency is reduced after STN DBS, however, available data suggests that DBS therapy may not change sleep fragmentation or RBD (39, 50, 51, 54, 55). Conflicting outcomes were seen in studies investigating the influence of DBS on RLS, (42, 47, 53) with some studies showing significant improvement in RLS scores while other studies reported no change and re-emergence of RLS post-DBS. These conflicting results could be due to different study methodology and future studies with similar methodology may help us to delineate this further. The effects of STN DBS on sleep-wake disturbances in PD have been attributed to improvement in motor symptoms, reduction in dopaminergic medications, and neuromodulation of basal-ganglia circuits affecting sleep physiology (39, 55, 67). STN DBS effectively improves motor symptoms and improves nocturnal mobility, which contributes in improving sleep quality. STN DBS may also be potentially modulating sleep-wake centers. However the exact neuromodulatory effects of STN DBS on sleep physiology is unknown and is further complicated by the fact that the therapeutic mechanism of DBS is still debated (30). There is currently very limited literature on effects of other DBS targets. DBS of GPi has shown improvement in sleep quality and efficiency, as well as reduced time awake and shortened REM latency. These findings were similar to the effects of STN DBS on sleep. More studies evaluating the effects of GPi DBS on sleep disturbances in PD are needed. PPN is another important target, which is being explored for its effect on gait and posture (68). Available studies examining effects of PPN DBS on sleep have shown that low frequency stimulation of PPN improves excessive daytime sleepiness, increase REM sleep, and improves sleep quality. Although the thalamus is a common target for DBS in movement disorders, the role of thalamic stimulation on sleep is not known.

Despite limitations in the current understanding of the pathophysiological role of DBS in sleep, this therapy has provided a great opportunity to study the role of neuromodulation for different non-motor symptoms. As sleep disturbances contribute to impaired quality of life, the DBS-mediated improvement in sleep is encouraging, particularly in light of the limited treatment options for sleep disturbances. Studying the effects of DBS at different sites can also help in understanding sleep pathophysiology. Future studies with larger samples, examining and comparing effects of stimulation at different targets on various aspects of sleep are needed.

## Expert statement

Sleep dysfunction is a disabling non-motor symptom experienced by the majority of patients with Parkinson disease (PD). Deep brain stimulation (DBS) is superior to best medical therapy (BMT) for improving the motor symptoms of PD. As the impact of non-motor symptoms on quality of life in PD has been increasingly recognized, the research community has turned attention to investigating the influence of DBS on these symptoms.

Studies on the influence of subthalamic nucleus (STN) DBS on sleep have consistently shown that this therapy improves subjective sleep quality. Many studies also suggest improvement in objective sleep measures, although different aspects of sleep architecture are improved in different studies. This may depend on several factors, including baseline sleep function, types of sleep complaints (i.e., nocturnal sleep dysfunction vs. daytime sleepiness), age, PD disease duration, location of DBS electrode within the nucleus, DBS settings, PD motor phenotype, or other factors. The improvement in sleep due to STN DBS could be related to improved motor symptoms, changes in medications, or alteration of neural circuits responsible for sleep. Additional research is needed to better delineate who might benefit most from the effects of DBS on sleep. Some of the research suggests that different approaches may be needed for individual patients and closed loop stimulation methodologies may help meet this need. Additional study is also needed to better define the influence of DBS at other targets, such as globus pallidus interna (GPi) and pedunculopontine nucleus (PPN), on sleep. This is an exciting area with promise and much to still be learned.

Harrison Walker, MD, University of Alabama at Birmingham, AL.

## Authors contributions

VS conception, execution, writing first draft, review, and critique. SS and SC in conception, review, and critique. AA in review and critique.

### Conflict of interest statement

The authors declare that the research was conducted in the absence of any commercial or financial relationships that could be construed as a potential conflict of interest.
